# Exposure of Prebiopsy Antibiotics Influence Bacteriological Diagnosis and Clinical Outcomes in Patients With Infectious Spondylitis

**DOI:** 10.1097/MD.0000000000003343

**Published:** 2016-04-18

**Authors:** Ying-Chih Wang, Chak-Bor Wong, I-Chun Wang, Tsai-Sheng Fu, Lih-Huei Chen, Wen-Jer Chen

**Affiliations:** From the Department of Orthopaedic Surgery, Keelung Chang Gung Memorial Hospital, Chang Gung University School of Medicine, Taiwan (Y-C W, C-B W, I-C W, T-S F); and Department of Orthopaedic Surgery, Linkou Chang Gung Memorial Hospital, Chang Gung University School of Medicine, Taiwan (L-H C, W-J C).

## Abstract

The benefit of prebiopsy empirical antibiotics for patients with infectious spondylitis and the effect on clinical outcome are not well known. This study assessed the impact of prebiopsy empirical antibiotics in patients with infectious spondylitis.

We retrospectively reviewed 41 adult in-patients with infectious spondylitis who received percutaneous endoscopic debridement and drainage (PEDD) at a tertiary care hospital from August 2002 to August 2012. The average patient age was 55.2 years old and causative bacteria were identified in 32 out of 41 biopsy specimens (78.0%) via the PEDD procedure, which has good diagnostic efficacy comparable to open biopsy.

Seventeen patients (41.5%) received prebiopsy empirical antimicrobial therapy, and these patients were less likely to have positive cultures than those who did not receive preoperative antibiotics (64.7% vs 87.5%, *P* = 0.04). Patients with positive cultures had a better infection control rate (78.1% vs 67.7%) and were less likely to undergo subsequent open surgery. Patients given preoperative antibiotics were more likely to need subsequent open surgery (35.3% vs 16.7%, *P* = 0.02). From multivariate logistic analysis showed age at diagnosis to be an independent risk factor for the need of further surgery. There were no major complications following the PEDD procedure, except 2 patients had transient paresthesia in the affected lumbar segments.

Prebiopsy empirical antibiotic therapy was associated with lower positive culture rate and an increased need for subsequent open surgery. Patients with positive cultures were more likely to have initially adequate treatment, better infection control, and better clinical outcome.

## INTRODUCTION

Infectious spondylitis is basically a medical disease. Parenteral antibiotics are the mainstay of treatment for infectious spondylitis.^1,2^ Antibiotic treatment must start early to prevent disease progression and development of potentially irreversible neurologic deficits. Identifying the offending pathogen is critical for the administration of appropriate antibiotics.^3–5^ However, antibiotic selection is often guided by empirical evidence because clinicians want to begin treatment as soon as possible. In fact, clinicians routinely prescribe antibiotics for the treatment of infectious spondylitis with no evidence of the causative pathogen or its antibiotic sensitivity.^6,7^

Empirical antibiotics work in some but not all patients with infectious spondylitis. When pharmacological treatment fails, the disease often progresses to a more advanced and difficult-to-manage condition. As the patient may be too ill for extensive open surgery, which is suffering and may lead to wound problems, neurovascular injury, and some inevitable anesthesia complications, especially in patients who are immuno-compromised or with multiple comorbidities. Some reports indicated that administration of prebiopsy antibiotics had a negative effect on microbiologic diagnosis,^8–11^ but others indicated no such effect.^12^ No literatures have investigated the relationship between prebiopsy antibiotics and clinical outcomes in patients with infectious spondylitis.

Nowadays, there are several newly developed minimally invasive procedures for spinal disorders.^13,14^ In particular, percutaneous endoscopic debridement and drainage (PEDD) has both good therapeutic and diagnostic value, which comparable to open surgery in patients with spinal infections.^15–17^ The purpose of this study was to confirm the impact of prebiopsy empirical antibiotic treatment on the bacteriological diagnosis and clinical outcome of patients with infectious spondylitis who underwent the PEDD procedure.

## MATERIAL AND METHODS

### Study Population

This retrospective study was approved by the institutional review board of Chang Gung Memorial Hospital. We retrospectively enrolled 41 patients with infectious spondylitis who underwent PEDD from August 2002 to August 2012 at Chang Gung Memorial Hospital. Patients with infections associated with neurological deficits were excluded. Infectious spondylitis was diagnosed clinically based on clinical symptoms, elevated erythrocyte sedimentation rate (ESR) and C-reactive protein (CRP) level, radiograph, and magnetic resonance imaging results. All patients presented with intractable back pain and required narcotics for pain control and bed rest. Seventeen patients (41.5%) received empirical antibiotic treatment before the PEDD procedure (vancomycin or cefazolin + gentamicin). These 41 patients were divided into 2 groups: those who received prebiopsy antibiotics and those who did not.

### Surgical Procedures

The PEDD procedure is used to debride and cultivate causative microorganisms, as described in our previous study.^15^ It is performed via a posterolateral percutaneous approach using the Yeung Endoscopic Spinal System (Richard Wolf GmbH, Knittlingen, Germany) under local anesthesia and conscious sedation. All patients were positioned in prone position. A spinal needle was inserted directly into the infected region under guidance of intraoperative fluoroscopy. The abscess within the lesion was aspirated and sent for culturing. Discectomy forceps were inserted to debride necrotic tissues. The collected specimens were subjected to aerobic and anaerobic cultures, tuberculosis culture and polymerase chain reaction, fungal culture, and histopathological examinations.

After biopsy and debridement, irrigation was performed using normal saline, after which the intradiscal lesion was endoscopically examined. Finally, a drainage tube (diameter: 3.2 mm) was inserted into the debrided disc space and connected to a negative-pressure pump (Hemovac; Zimmer, Dover, OH). All tubes were left in place until the drainage stopped or declined to less than 10 mL/day for 3 consecutive days. After the operation, patients were allowed to walk while wearing a Taylor brace.

### Outcome Measures

These 2 groups (with prebiopsy antibiotics vs without prebiopsy antibiotics) were compared in terms of clinical symptoms, serological tests (white blood count, CRP, and ESR), and imaging results to determine whether continued conservative treatment was sufficient or open surgical intervention was required with a minimal 2 year follow-up. Preoperative and postoperative pain were evaluated by a visual analog scale (VAS, range: 0–10). We also compared preoperative CRP with CRP at 1 month after surgery, and preoperative ESR with ESR at 2 months after surgery. The rate of positive cultures was also compared in these 2 groups. If a patient needed a 2nd open surgery or died, we defined it as a failed treatment.

### Statistical Methods

Statistical evaluation was performed using the SPSS 13.0 software (SPSS Inc, Chicago, IL). A comparison between the 2 groups was conducted using the independent *t* test. Fisher exact test was used to access the difference of successful culture rate and the need of secondary open surgery between 2 groups. *P* < 0.05 was considered as a significant difference. Multivariate logistic regression was also performed on these variables, including age, gender, preoperative antibiotics, and negative culture result, to investigate the independent association with the rate of further surgery. All patients were followed up for at least 2 years after undergoing the PEDD procedure.

## RESULTS

### Patient Characteristics

We retrospectively enrolled 41 patients with infectious spondylitis who underwent PEDD, 17 of whom received prebiopsy antibiotics and 24 of whom did not (Table 1). There were 29 males and 12 females, and the average age was 55.2 years (range: 28–88 years). The spinal regions of infection were L1–L2 in 2 patients, L2–L3 in 6 patients, L3–L4 in 5 patients, L4–L5 in 16 patients, and L5–S in 12 patients. All patients had back pain, 14 patients had fever, and 11 patients had pain radiation to a leg as initial symptoms. Among patients who received prebiopsy antibiotics, 4 patients had type II diabetes, 2 had liver cirrhosis, 2 had renal failure, 1 had congestive heart failure, and 1 had acquired immune deficiency syndrome. Among patients who did not receive prebiopsy antibiotics, 4 patients had diabetes, 4 had liver cirrhosis, 3 had renal failure, 2 had congestive heart failure, and 1 had acquired immune deficiency syndrome.

**TABLE 1:**
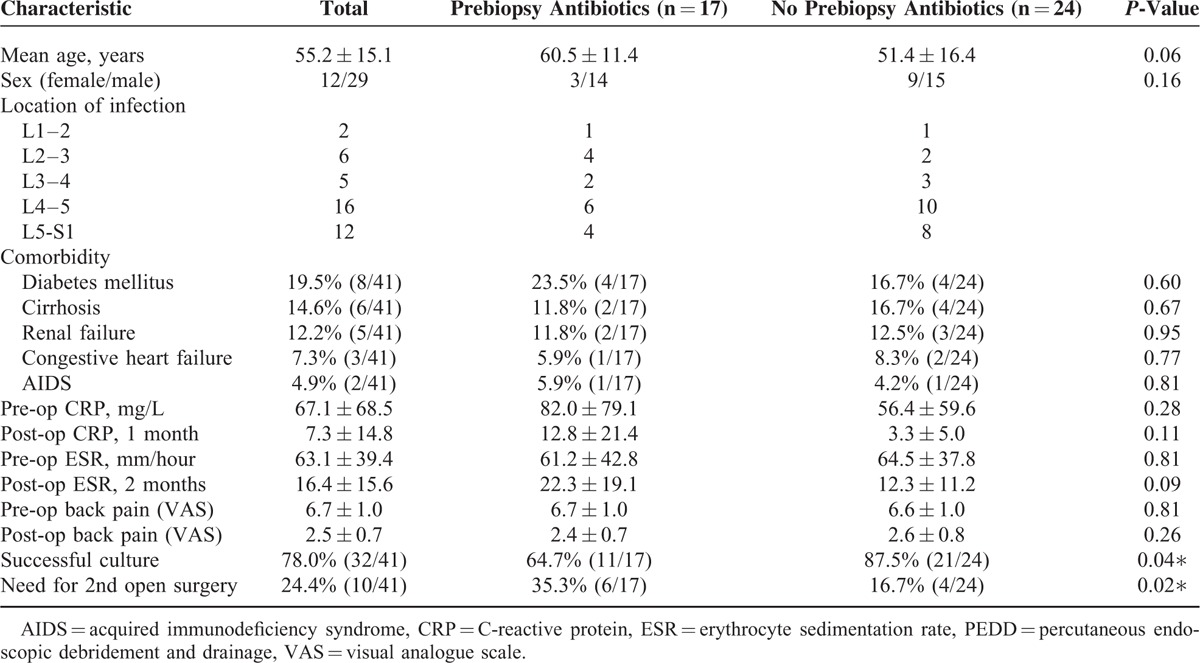
Characteristics of Patients With Infectious Spondylitis Who Received or Did Not Receive Antibiotics Before the PEDD Procedure (n = 41)

### Microbiological Results

We successfully isolated the causative organism in 32 patients (78.0%) following the PEDD procedure (Table 2). Nine patients were infected with *Staphylococcus aureus* (5 with an oxacillin-resistant strain and 4 with an oxacillin-sensitive strain), 6 patients with *Mycobacterium tuberculosis*, 3 with *Enterococcus faecalis*, 3 with *Escherichia coli*, 2 with *Streptococcus viridans*, 2 with gram-positive bacilli, 2 with *Candida albicans*, and the other 5 with *Streptococcus bovis*, *Prevotella*, *Pseudomonas aeruginosa*, *Peptostreptococcus micros*, or *Klebsiella pneumoniae*. Among the 32 cultures that exhibited growth, *S. aureus*, which typically exists on human skin, was the most common pathogen (28.1%), followed by *M. tuberculosis* (15%).

**TABLE 2:**
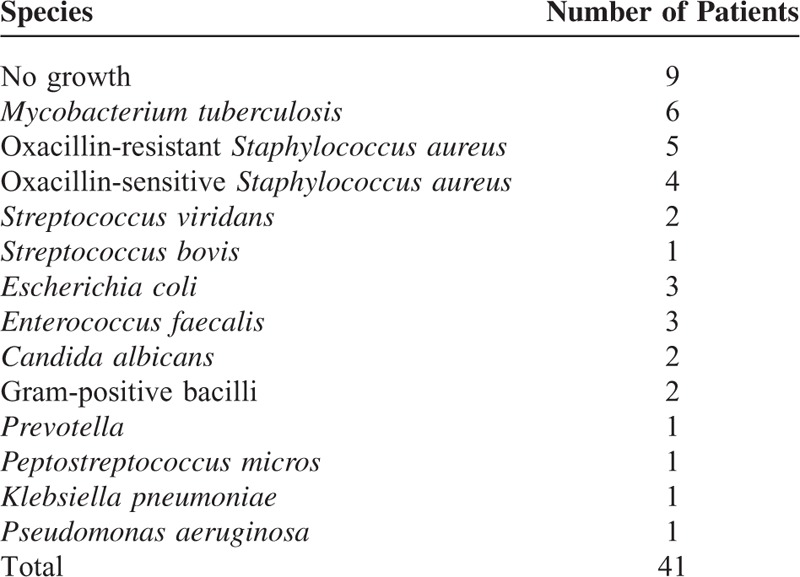
Microbiological Test Results of Patients With Infectious Spondylitis

A regimen consisting of systemic antibiotics, antituberculous agents, or antifungal agents was administered according to sensitivity studies for each identified pathogen. In the entire study population, patients with positive culture results had a higher infection control rate than those without positive culture results (78.1% vs 66.7%, Table 3). In other words, positive culture results reduced the need for subsequent open surgery.

**TABLE 3:**
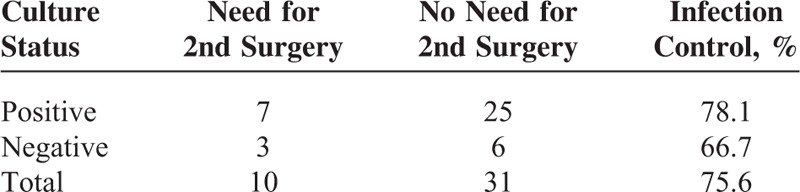
Effect of Microbiological Culture on Control of Infection in Infectious Spondylitis

Seventeen patients received empirical antibiotics therapy before the PEDD procedure, and their positive culture rate was 64.7% (11/17). In patients without empirical antibiotics therapy, their positive culture rate can reach to 87.5% (21/24). Without preoperative antibiotics therapy, the positive culture rate is statistically significant higher (Table 1).

### Clinical Outcomes

**FIGURE 1:**
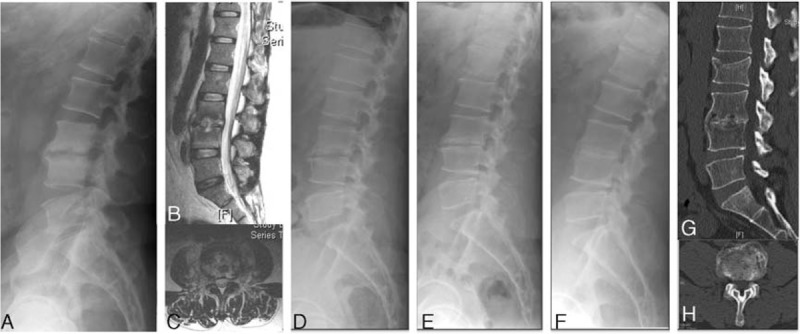
A 35 year-old man presented with severe pain in the lower back and leg, and was diagnosed with an L3–4 methicillin-resistant *Staphylococcus aureus* infection. (A–C) Preoperative plain radiograph and magnetic resonance imaging showed L3–4 disc and endplate destruction. (D) Plain radiograph at 1 month after surgery. (E) Plain radiograph at 1 year after treatment showed endplate sclerosis without further destruction. (F–H) Plain radiograph and computed tomography at 5 years after treatment showed well-maintained lumbar lordosis.

A total of 31 patients (75.6%) reported satisfactory relief from back pain after the PEDD procedure and required no further open surgery (Figure 1). For patients who received prebiopsy antibiotics, the average VAS for low back pain was 6.7 ± 1.0 before surgery and 2.4 ± 0.7 at 1 month after surgery, CRP was 82.0 ± 79.1 mg/L before surgery and 12.8 ± 21.4 mg/L at 1 month after surgery, ESR was 61.2 ± 42.8 mm/h before surgery and 22.3 ± 19.1 mm/h at 2 months after surgery (Table 1). For patients not given prebiopsy antibiotics, the average VAS for low back pain was 6.6 ± 1.0 before surgery and 2.6 ± 0.8 at 1 month after surgery, CRP was 56.4 ± 59.6 mg/L before surgery and 3.3 ± 5.0 mg/L at 1 month after surgery, ESR was 64.5 ± 37.8 mm/h before surgery and 12.3 ± 11.2 mm/h at 2 months after surgery. Thus, these 3 clinical parameters all improved after the PEDD procedure in each group, but there were no statistically significant differences between the groups.

Ten patients (24.4%) received further open surgery, due to either intractable back pain, mechanical spine instability, or persisting infection. Two patients died. A 78 year-old female in the prebiopsy antibiotics group who had a *C. albicans* infection died 8 months after surgery due to flare up of COPD and extensive comorbidities (pneumoconiosis, pulmonary tuberculosis, and congestive heart failure) in. A 52 year-old female with hypertension, chronic renal failure and treated by regular hemodialysis, congestive heart failure, and an oxacillin-resistant *S. aureus* spinal infection died 6 months after a 2nd anterior debridement surgery. There were no major surgery-related complications, although 2 patients had transient paresthesia in the dermatome of the affected lumbar segment.

Patients who received prebiopsy antibiotics were more likely to need subsequent open surgery than those not given prebiopsy antibiotics (35.3% vs 16.7%, *P* = 0.02, Table 1). The subsequent open surgery procedures included anterior debridement and autograft interbody fusion with an autogenous tricortical iliac bone graft with or without posterior instrumentation for mechanical instability.^18,19^

From multivariate logistic analysis, older age at diagnosis is the independent risk factor for the need of further surgery (adjusted odds ratio [OR], 1.19, Table 4). Negative culture result, male, and given preoperative antibiotics had higher adjusted OR of further surgery (3.47, 2.47, and 1.14, respectively), however, not reach to statistical significance.

**TABLE 4:**
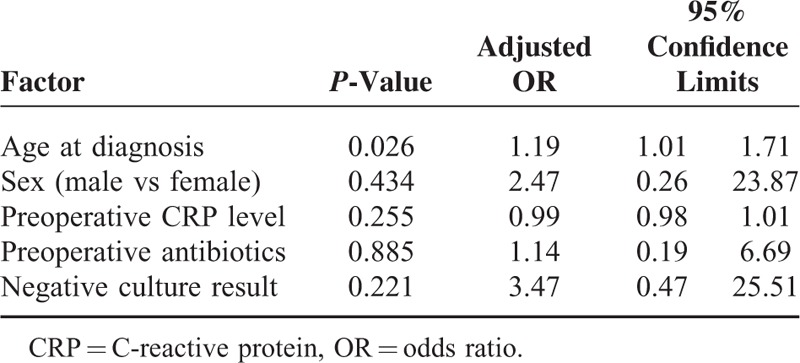
Multivariate Logistic Regression Analysis and Adjusted ORs

## DISCUSSION

The impact of prebiopsy empirical antibiotic treatment on the bacteriological diagnosis and clinical outcome of patients with infectious spondylitis is controversial. The results of the current study show that prebiopsy empirical antibiotic treatment was associated with a lower rate of microbiological diagnosis in such patients. In particular, patients without prebiopsy antibiotics had a positive culture rate of 87.5%, but those given prebiopsy antibiotics had a positive culture rate of only 64.7%. Patients who had positive cultures were more likely to receive initially adequate treatments, had a higher infection control rate, and had better clinical outcomes. Patients given prebiopsy antibiotics were more likely to need subsequent open surgery than those not given prebiopsy antibiotics (35.3% vs 16.7%, *P* = 0.02). Besides, multivariate logistic analysis showed age at diagnosis to be an independent risk factor for the need of further surgery.

Many clinicians treating patients with infectious spondylitis administer empirical antibiotics before biopsy.^4,6,7^ Some researchers recommend a combination regimen (ciprofloxacin + clindamycin) because this provides control of Staphylococci and gram-negative species. However, this prebiopsy antibiotic regimen may reduce the ability to isolate the causative organism and determine its antibiotic sensitivity.^9–11^ Thus, our departmental policy recommends against initiation of antibiotics prior to biopsy in patients with infectious spondylitis. However, 17 of the 41 patients examined here received prebiopsy antibiotics because they were transferred from other departments or other hospitals. Marschall et al^12^ retrospectively examined 91 patients with hematogenous vertebral osteomyelitis who received open biopsy or needle biopsy, and found that open biopsy was associated with a positive biopsy result but that prebiopsy antibiotic therapy was not associated with negative culture results. However, these patients had greatly varying severity of disease and 2 biopsy methods were used. We examined patients without neurological deficits and used a single minimally invasive method (comparable to open biopsy) and found that prebiopsy use of empirical antibiotic therapy led to a lower culture rate (87.5% vs 64.7%, *P* = 0.04). Kim et al^10^ studied patients with microbiologically and clinically diagnosed vertebral osteomyelitis and also found that prior antibiotic use, especially use of long duration, was strongly associated with negative culture results. In agreement, Rankine et al^11^ reported that empiric antibiotic therapy before computed tomography (CT)-guided or fluoroscopy-guided percutaneous biopsy reduced the positive culture rate from 40% to 25%.

Kollef et al^20^ traced 2000 consecutive patients who required admission to the medical or surgical intensive care unit (ICU) and found that 169 (8.5%) of infected patients received inadequate antimicrobial treatment. They also had 2 other major findings. First, prior administration of empirical antibiotics (OR = 3.39, *P* < 0.001) and presence of a bloodstream infection (OR = 1.88, *P* = 0.003) were independently associated with the administration of inadequate antimicrobial treatment. Second, in-hospital mortality of patients receiving inadequate antimicrobial treatment was significantly greater than that of other patients (52.1% vs 12.2%, relative risk [RR] = 4.26, *P* < 0.001). In our study, prebiopsy use of antibiotics was also associated with poorer clinical outcome, and these patients had a greater need for a subsequent open surgery. Prior administration of an inappropriate antibiotic may lead to colonization of bacteria that are resistant to that entire class of antibiotics. More importantly, colonization with antibiotic-resistant pathogens may increase the risk of subsequent infection with these same highly virulent microorganisms. The current study indicates that empirical antibiotics should not be routinely administered to patients with spinal infections who are scheduled to receive biopsies or surgical procedures. As empirical antibiotic therapy is perforce broad spectrum and unfocused, it should be delayed until results of blood cultures or when appropriate biopsy specimens are available unless a patient is severely septic, critically ill, or neurologically compromised.

CT-guided needle biopsy is currently the most common minimally invasive method for obtaining specimens in patients with spinal infections, but the rate of successful microbiological identification can vary from 32% to 91%.^3,21^ Yang et al^3^ reported that the rate of successful microbiological identification was better for percutaneous endoscopy than CT-guided biopsy (18 of 20 [90%] vs 15 of 32 [47%]). Percutaneous endoscopy is a minimally invasive method that can be used both for diagnosis and therapy. This method has numerous advantages for patients with infectious spondylitis: it can provide immediate relief from back pain while preserving the motion of the affected vertebral segment; it provides observations of the lesion site pathology directly; and it allows drainage of pus and collection of sufficient specimens for accurate bacteriological diagnosis. Based on culture results, clinicians can prescribe appropriate antimicrobial therapy so that secondary surgery is not necessary. Yang et al used percutaneous endoscopy to identify causative bacteria in 19 of 21 (90.5 %) biopsy specimens from spondylitis patients with advanced lumbar infectious and successfully treated these patients without surgery-related complications or neurological deficits.^22^ The positive culture rate of the PEDD procedure is comparable with that from open biopsy. Ito et al^17^ used the same posterolateral endoscopic technique and successfully treated 15 patients with pyogenic spondylitis in the thoracic or lumbar spine. PEDD is a minimally invasive procedure for diagnosis and therapeutic treatment for spinal infections. It is an effective alternative and should be considered prior to extensive spinal surgery particularly for patients with early-stage spinal infection or serious medical conditions.^15^There are some limitation in our study, our cases are retrospective and not randomized and the sample size is relatively small. Further large sample size study still needs to execute.

## CONCLUSION

Our study of patients with infectious spondylitis indicated that prebiopsy empirical antibiotic therapy had a negative effect on microbiological diagnosis. Patients with positive cultures had a higher rate of infection control and better clinical outcome. Thus, we advocate no prebiopsy antibiotics for patients with infectious spondylitis who are scheduled for imminent biopsy or surgical treatment, because it may affect the culture results and clinical outcome. With improved endoscopic instruments and techniques, spinal infections can be successfully treated by minimally invasive percutaneous endoscopic debridement.
